# Exploring the Impact of Parkinson’s Disease on Marital Relationships

**DOI:** 10.3390/nursrep16040113

**Published:** 2026-03-31

**Authors:** Pardis Momeni, Elisabeth Winnberg

**Affiliations:** Department of Health Care Sciences, Marie Cederschiöld University, P.O. Box 11189, SE-100 61 Stockholm, Sweden; elisabeth.winnberg@gmail.com

**Keywords:** Parkinson’s disease, marital relationship, nursing, qualitative method

## Abstract

**Background/Objectives**: Parkinson’s disease (PD) is a progressive neurodegenerative disorder that affects both motor and non-motor functioning, leading to increasing dependency and long-term psychosocial consequences. As the disease progresses, partners often assume caregiving roles, resulting in shifts in responsibilities, communication patterns, and emotional dynamics within marital relationships. The aim of this study was therefore to explore the impact of Parkinson’s disease on marital relationships. **Methods**: A qualitative interview study with a retrospective design was conducted. Six couples were recruited through a movement disorders clinic and a lay organization in Sweden. Semi-structured, face-to-face interviews were conducted separately with each partner. Interviews were transcribed verbatim and analyzed using conventional content analysis with an inductive design. **Results**: Four main themes emerged: managing the disease together in partnership, nurturing the relationship, facing marital hardship, and planning an uncertain future. Couples who adopted a positive and pragmatic outlook, shared responsibilities, and maintained open communication seemed to be better able to manage the disease. Engaging in joint activities and reciprocal communication strengthened emotional closeness. In contrast, changes in roles, emotional distress, loss of intimacy, and communication avoidance challenged relationships. Thinking about the future evoked feelings of ambivalence, as couples balanced uncertainty with a need for security. **Conclusions**: Parkinson’s disease affects marital relationships, reshaping roles, emotional bonds, and future perspectives. The ability of nurses to address both partners’ needs and promote communication and shared coping strategies is essential to strengthening couples’ well-being.

## 1. Introduction

Parkinson’s disease (PD) is a complex neurodegenerative disorder affecting approximately 6 million people worldwide [1]. It is the fastest-growing neurological disorder in terms of prevalence, disability, and mortality. The incidence of PD increases markedly with age, particularly among individuals aged 65 years and older [2]. Although PD is primarily characterized as a movement disorder, most patients also experience non-motor symptoms (NMS) that can affect nearly all organ systems. Motor symptoms include bradykinesia, muscular rigidity, resting tremors, as well as postural instability and gait abnormalities [3,4,5]. NMS are diverse and include neuropsychiatric manifestations, such as depression, anxiety, and sleep disturbances, as well as autonomic and cognitive dysfunction, including dementia [3,4,5,6]. Similar to motor symptoms, NMS can fluctuate in their response to dopaminergic treatment. During so-called OFF or wearing-off periods, the effects of medication diminish, leading to the return or worsening of symptoms, which can be very disabling, contributing to the overall unpredictability of daily life [7]. Furthermore, both motor symptoms and NMS have been shown to negatively affect health-related quality of life (HRQoL) in persons with PD (PwPDs) [8]. As PD progresses, PwPDs typically experience a gradual decline in their ability to perform activities of daily living, resulting in an increasing need for assistance over time [5].

Eventually, many PwPDs become dependent on their partners for assistance or help with daily activities, with partners frequently assuming the role of primary informal caregivers. This increasing caregiving responsibility can become challenging and burden partners [9,10] and may also limit partners’ opportunities for an unrestricted social life, both with and independent of the PwPD [11]. It is also known that partners are at risk of poor health due to this increased strain [12]. However, PD does not only affect the PwPDs and caregivers individually but may also have a negative impact on the quality of the relationship, which has been suggested to mediate the burden of caregiving [13]. Mutuality in a relationship includes dimensions such as love and affection, shared pleasurable activities and reciprocity (mutual giving and taking) and has been suggested to be a protective factor against caregiver strain in PD [14,15,16]. Furthermore, Karlstedt et al. [17] indicated that increasing mutuality mediates the effect of motor symptoms and NMS on HrQoL in PwPDs. A review by Glover et al. [18] found that couples affected by PD experience disrupted roles and responsibilities due to declining functional ability. Communication and shared activities decreased, and partners often reported grief, burden, and social isolation linked to cognitive and behavioral changes. Although there is some qualitative research in this area, the number of studies is still limited. Taking this into consideration, we therefore wanted to explore the impact of PD on marital relationships.

## 2. Materials and Methods

### 2.1. Design

This was a qualitative interview study.

### 2.2. Data Collection

PwPDs were recruited by purposive sampling from the movement disorders clinics at the Karolinska University Hospital, Stockholm and Huddinge, Sweden (three couples) and through an advertisement in the journal of the Swedish Parkinson’s Disease Association (three couples). Inclusion criteria were being diagnosed with PD by a specialist, living with a partner before and after the diagnosis, and both the PwPD and the partner were willing to participate. Six couples were invited to participate and no one declined. (Table 1). The PwPDs were diagnosed 7–28 years ago (mean 15 years) and were aged 65–82 years (mean 71 years) at the time of the interview. All PwPDs experienced on–off fluctuations but received no advanced therapy such as Deep Brain Stimulation (DBS). They were in stage 4 or 5 (out of five stages) of the disease according to Hoehn & Yahr [19]. None of them had a cognitive decline. The partners were aged 66–78 years (mean 72 years) at the time of the interviews, and their relationships had been ongoing for 31–60 years (Table 1).

Data was collected through semi-structured face-to-face interviews with a retrospective design [20]. An interview guide was used (Appendix A) focusing on the couple relationship, both before the PD diagnosis and the time since. The interview guide was not pilot tested before the interview. The interviews were performed during 2015–2018 with the couples’ written informed consent (Appendix B) and held either at the couple’s home or at the clinic. An external PD specialist nurse (RN, PhD) conducted the interviews with the patients in all but one case. She was partly involved in three of the PwPDs’ care. The second author (EW), a specialist nurse in Huntington’s disease (RN, Associate Professor), conducted the interviews with the partners in all but one case. She was not involved in the PwPDs’ care. All 12 participants were interviewed separately; the interviews lasted between 39 and 77 min and were tape-recorded. No field notes were taken during the interviews. The tape-recorded interviews were of good quality, allowing the material to be clearly heard and accurately transcribed. Interviews were transcribed verbatim, resulting in approximately 190 single-spaced typewritten pages. The interviews were managed and analyzed electronically. The digital materials were stored securely according to the data security policy at the university. Access was restricted to the authors of the study only.

The sample was specific to the study’s aim, and the interviews provided rich and detailed data. Data saturation was considered reached after the 12 interviews, as no new themes or relevant information emerged during the later stages of the analysis. The quotations in Swedish by the participants were translated into English by a professional translator. No back-translation was performed, but the translations were reviewed by the authors against the original to ensure accuracy. Idiomatic and emotion-laden expressions in participants’ quotes were carefully translated to preserve their intended meaning and emotional tone rather than translated word-for-word. The names of the participants in the quotations have been changed to maintain anonymity.

### 2.3. Data Analysis

The interviews were analyzed using conventional content analysis with an inductive approach [21], which was performed in four steps, which comprised the coding process. Firstly, the transcripts were thoroughly read by the authors independently to gain a holistic overview. Quotations relevant to the aim of the study were identified, marked in the transcripts and labeled with a content heading. Secondly, these quotations were discussed, and meaning units were identified and organized into a table. In the third step, we analyzed the quotations from each couple independently, looking for differences and similarities in the transcripts between the group of partners and the group of PwPDs. These were discussed and the coding process table was extended with a column for codes. In the fourth step, mind-map diagrams were used to visualize how codes relate to each other and how the findings were condensed into themes and subthemes. During all four steps, the quotations were discussed and the results revised until agreement was achieved (Table 2). Our analysis resulted in four main themes and nine sub-themes. The fourth theme does not contain any subthemes. The themes are summarized in Table 3 with quotation examples.

### 2.4. Trustworthiness

The concepts for trustworthiness in qualitative research are credibility, dependability, confirmability and transferability [22]. To ensure credibility, researchers must acknowledge personal biases and preconceptions throughout the analysis process (reflexivity). This was continuously addressed during the analyses by persistent observation. The authors had no previous clinical experience in PD, and instead had experience with families with Huntington’s disease and geriatric nursing; therefore, we discussed our personal biases based on other clinical experiences outside of PD, and challenged each other to keep an open-minded attitude towards the data. Dependability verifies that the study findings are consistent with the collected data [22]. It was established through the authors’ equal engagement in the analyses with repeated readings of the transcripts, independent coding and recurrent discussions of the emerging codes and themes. An audit trail was used throughout the process of analysis, consisting of documentation of the quotations in tables for the evolving codes, themes and subthemes. For confirmability, to maintain the objectivity of the findings [22], peer debriefing was practiced. The PD specialist nurse involved in the interviewing process was used as a “critical friend” by reading the interview transcripts and was part of the discussions on themes and subthemes. Finally, by providing detailed information about the participants (Table 1) and the methods of data collection (thick descriptions), readers are enabled to assess the transferability of findings to other contexts.

## 3. Results

The results showed that managing the disease together in partnership and unity, despite the challenges couples faced now and those that they knew were ahead, meant trying to have a positive outlook on life and their current situation. The life that they once had, and the roles within the relationship that they were used to, were forever changed because of PD and the adjustments that it requires. Some couples could manage the changes thanks to their strength, togetherness, and the internal and external support they received, as well as joint efforts made to enable them to have time alone, especially the partner. But for some, the changes were simply too much to handle, and there was instead a wish for solitude that was notable, which resulted in feelings of hopelessness and a lack of communication. Talking about the future was a bit like talking about the elephant in the room; some avoided it because they knew what was coming, and others felt safer if there was a plan going forward.

### 3.1. Theme: Managing the Disease Together in Partnership

During the interviews, reflections on dealing with the disease together were frequently noted. The unity between the couples and their personal inner strengths led them to be better at handling the hurdles of PD. The following subthemes emerged: (i) A positive attitude towards life, (ii) having a pragmatic outlook on life, (iii) providing support and enabling personal time for their partner.

#### 3.1.1. Subtheme: A Positive Attitude Towards Life

With a positive attitude, problems in daily life were easier to handle. It was described as a way of seeing the bright side of life. It was easier because they realized the alternative would not benefit them either.

“*I’m a positive person and feel like, ‘Yeah yeah, we’ll sort this out. What will be will be. We’ll make the best of it.’ Then it’s much easier to… it sounds strange, but it’s much easier to handle everyday life with that attitude than to wake up in the morning and think everything is awful.*”

Christina, PwPD (age 65, couple 3)

“*No one understands how we can live the way we do. ‘But how are you holding up?’ ‘I’m fine!’ ‘Oh? How is that possible?’ ‘Well, that’s the easiest way.’ It’s much easier to be happy than to be sad. Sure, it’s sad that we can’t do this or that, but you can’t have everything. I still have so many other things.*”

Susanne, partner (age 75, couple 6)

“*If you were to get depressed, that wouldn’t help much. It doesn’t solve any problems. It’s better to look on the bright side.*”

Carl, PwPD (age 75, couple 6)

#### 3.1.2. Subtheme: Having a Pragmatic Outlook on Life

A pragmatic view of how to handle problems related to the disease emerged. Problem-solving strategies appeared to be an effective way of coping with the disease. The pragmatic outlook was a joint understanding and effort when dealing with the progression of PD.

“*Yep, we just had to get on with it. And that’s what we’ve done our whole lives with everything. ‘Alright, this is how it is. Then we’ll do it this way.’*”

Susanne, partner (age 75, couple 6)

“*My happiness is the same whether I’d got Parkinson’s or not, but I don’t think it really… it may sound strange, but I don’t think it matters that much. I had to modify my interests because it wasn’t possible to do everything.*”

Stephan, PwPD (age 68, couple 5)

“*We’ve decided that we’re going to handle this together. // I think that… that you must decide at some point who is in charge, is it me or my illness, and as long as you’re the one in control… then you… then that’s a small victory.*”

Christina, PwPD (age 65, couple 3)

#### 3.1.3. Subtheme: Providing Support and Enabling Personal Time for Their Partner

Partner support meant being involved but also providing the partner with opportunities to have some personal time. Partners expressed the importance of having time alone away from their PwPD. Partner support helped them deal with PD and uphold the relationship. Gaining new knowledge and perspectives by being involved in the care of the PwPD also led to personal development and skills that could be used in other contexts of life.

“*I was mostly there as an observer and as a second memory, so that I could understand when she couldn’t take in all the information. It was incredibly important for me to be there and hear everything… I’ve started taking a great interest in Parkinson’s disease… sometimes I ask about things that I maybe didn’t ask about before because I used to take things for granted… I’ve tried to learn that… that even men can talk to each other, because often we’re like, ‘well, that’s nothing you need to talk about,’ and so on.*”

Alfred, partner (age 70, couple 3)

“*I went to a course last summer… four overnight stays… and we were thinking about how it would work with me being away for four days and so on. And she said, ‘I mean, we’ll work it out, you can… sign up,’ … yeah, something like that … Either someone could stay with her at home, or she could go and stay with a relative and get help. So, it was manageable.*”

Thomas, partner (age 70, couple 2)

“*But it’s very important that she gets to go on trips and experience things that I can’t take part in. Without it being interpreted as some sort of threat to our relationship. I can imagine that there are couples who quickly feel threatened by that. You don’t keep up in the same way.*”

Eric, PwPD (age 69, couple 1)

### 3.2. Theme: Nurturing the Relationship

Aspects related to the core factors that are needed to maintain a relationship were highlighted by the participants in different ways. Maintaining the quality of the relationship required work and taking care of each other. This generated two subthemes that were important in strengthening the relationship: (i) Doing activities together and (ii) reciprocal communication brings closeness in the relationship.

#### 3.2.1. Subtheme: Doing Activities Together

The importance of having common interests and finding activities to do together meant finding time and making time for such activities. This was important in nurturing the relationship.

“*Having activities to do together, I see that as fun, as long as you can agree on the content and extent of these. It’s also a way of building new roots that hold you together. Marriage is like having a business.*”

Eric, PwPD (age 69, couple 1)

“*We are reading a book aloud together, for example, taking turns and then talking about it. Yes, so… I feel that we talk much more with each other now than we used to.*”

Thomas, partner (age 70, couple 2)

#### 3.2.2. Subtheme: Reciprocal Communication Brings Closeness in the Relationship

The couples had all been married for many years and reported that PD had brought them closer in their relationship. Reciprocal communication about thoughts and needs was an important way to maintain emotional connection, gain new perspectives, and find possible ways to solve disagreements. They had to learn not to be afraid of having different opinions.

“*But I find it difficult to see what differences have come about from us retiring at the same time and so on. I feel that our relationship has become closer, that we talk much more with each other about different things, about what is happening and what’s going on. So, I think it is… both closer and perhaps warmer too.*”

Marie, PwPD (age 69, couple 2)

“*We can be sad together and we can laugh a lot together, and we have the same kind of humor, so yes, it works, it works well… you could say that it has become a bit deeper. You realize that life doesn’t run smoothly on rails, there are many sidetracks, and that you need to take care of each other. And I think both of us have realized how important it is to take care of what we have, so to speak, and not… not get caught up in lots of petty arguments like we maybe did before, especially when she was healthy and you could argue about some silly thing. But we don’t do that now—we put it aside.*”

Alfred, partner (age 70, couple 3)

“*I’ve known him since I turned 17, so we’ve basically grown up together. He is my husband and my best friend, as they so beautifully say. And we’ve always been able to talk about everything, even though it has been much harder for him. I chatter about everything. But he has had to learn to talk about feelings. I mean, we’ve taught each other because we’ve been together for so long, and that gives us an enormous sense of security. And marriage—no matter how good you think it is—is a lifelong job. Nothing becomes good on its own, whether you are healthy or ill. You must work on it and… and show each other love, every day, because otherwise it eventually falls apart.*”

Christina, PwPD (age 65, couple 3)

### 3.3. Theme: Facing Marital Hardship

Couples dealing with PD described challenges in their relationships. The routines and roles within the relationships had changed. Knowing that their partner had experienced an increased workload affected the PwPDs, as did their partners expressing distress.

#### 3.3.1. Subtheme: Shifting Relational Roles Can Lead to Emotional Distress

When living with PD, roles and responsibilities between couples can change over time. The results showed that partners were involved in the care of the PwPDs and the PwPDs were dependent on their partners to varying degrees. This change in roles and responsibilities could lead to emotional distress and an increased workload for the partner.

“*I had to keep an eye on him to make sure he took his medication. He started forgetting that too. Suddenly I had to shoulder much more… And it became stressful for me to keep track of him all the time… Now it’s more about checking that the pump is running, because if it stops, he becomes bedridden… And if there’s too much stimulation during the day, he gets stressed so easily, even by fun things… I can feel now that I can’t rely on him in the same way. He’s like a fly—he starts something, leaves it, moves on to the next thing, and forgets. So, I have to nudge him constantly…//I was a bit angry and annoyed about everything I suddenly had to do. He doesn’t drive anymore, and I feel like it’s affected many parts of our lives—I’m managing everything all the time…//I’ve realized that I’ve been quite dependent on my husband. He has taken care of so much. He has done so much with the children too, been a very present father. And caring towards his wife, taking on a lot of the load. I’ve been treated like a princess.*”

Cecilia, partner (age 66, couple 1)

“*I need to have him as backup all the time to get things done… I feel that he is very kind and very good at helping, and he’s good at cooking, but he doesn’t like cleaning, and that’s hard for me because then I have to tell him, and still… yes, and then he kind of pretends not to notice… But it’s interesting because you can see the difference. We thought we had an equal distribution of chores, doing things together in different ways, but I’ve realized that I’ve been the one taking care of the garden, the plants, and the cooking—that’s the easiest thing to share, even though I get irritated.*”

Marie, PwPD (age 69, couple 2)

“*The difference is that she now needs to take over things that I normally used to do. But there are still things that I plan and decide about, too.*”

Michael, PwPD (age 82, couple 4)

#### 3.3.2. Subtheme: Partners’ Desire for Solitude

During the hardships couples were faced with, having time alone from each other to do whatever they wanted was important to be able to keep going on.

“*I feel stressed all the time, I can’t relax, and I feel a strong need to be alone. But I’m not alone as much as I want to be.*”

Helen, partner (age 75, couple 5)

“*I really feel a strong urge to be by myself a lot. I’m quiet. Eric finds it very difficult that I have such a need to be silent and not talk all the time. I get my alone time every evening when I meditate. I have a glass of red wine and if the weather is nice, I sit and watch the sunset, and Eric isn’t allowed to disturb me. During the winter, I sit by the fireplace with a glass of red wine. That’s when I think about and reflect on the day. I also read a lot…//So I feel… yes, I imagine the thought of being by myself, living here alone. And it doesn’t feel strange. And sometimes it even feels nice. I’m admitting this to you now. I’m admitting it to myself, but I haven’t admitted it to anyone else… You’re not supposed to be this cruel, and it’s hard to say all this.*”

Cecilia, partner (age 66, couple 1)

#### 3.3.3. Subtheme: Lack of Reciprocal Communication and Mutual Understanding 

Some couples described a lack of understanding or communication avoidance that led to unresolved issues, emotional distance, and loneliness in the relationship. For some, couples therapy was one way to gain a greater understanding of each other’s thoughts and needs.

“*I know what I want to say and I know what I mean to say, but I can’t find the words, and then I get irritated with Alfred when he tries to fill in what’s missing, you know? It’s a bit like with someone who stutters—you’re not supposed to help, and I don’t want help either. ‘Shall I help you with your jacket?’ he says. ‘I can do it myself.’ ‘Let it take time, but I want to do it myself. I don’t want to be that helpless, you know?’ That’s how I feel. No, I really don’t want that.*”

Christina, PwPD (age 65, couple 3)

“*She is still young and energetic, while I’m aging much faster. I would love to talk about old memories, but she thinks we’re ‘done with that,’ and I can tell she gets a bit tired of that. I’ve said things so many times before… I can’t add to the stock of stories and experiences anymore like I used to. When you’re out and active, you always have something new to tell. She feels that we’ve already talked enough about those things… I felt that we weren’t getting closer, that we couldn’t sort this out together anymore. And we had to do something because it felt ominous—I truly think we might have ended up in a separation crisis sooner or later. It was incredibly fortunate that we got to see a therapist. We really love her, she’s wonderful. She asks such thoughtful questions, gently. And then we bring up things that we wouldn’t have done on our own, Cecilia and I. Almost every time we leave, we feel like newly washed diamonds—things that I hadn’t seen before, they shine beautifully and you understand more.*”

Eric, PwPD (age 69, couple 1)

“*When you’re struggling a bit and are lying awake at night unable to sleep, it’s not something you can just shake off the way you once could. It’s hard, but I can’t go and wake her up to start talking about it. No, no—then you’re alone with your thoughts. Yes, I think she… I think she has similar thoughts. She has said something once or twice that made me understand she thinks the same way. I don’t remember exactly what she said, but something that made me realize she’s had the same thoughts about death and long-term care and all that.*”

Stephan, PwPD (age 68, couple 5)

#### 3.3.4. Subtheme: Loss of What Once Was

Sorrow was described by the partners as a loss of a life that was supposed to take another direction. The sorrow of losing a part of their partner with PD was described in different ways. But also, a loss of intimacy was described, both because of PD and age.

“*Yes, you grow into things, you really do. But it was also sorrowful that he wasn’t the rock in my life. I think it has been… a dependence because he is the greatest love of my life. It’s a good kind of dependence…//So it’s not only negative. He is a very big part of my life. It has been very sad.*”

Cecilia, partner (age 66, couple 1)

“*And I thought I would have a partner that I could do fun things with. But it didn’t quite turn out that way.*”

Helen, partner (age 75, couple 5)

“*We have our age against us, both of us. I talk to the nurse about it, that sex has faded away. My partner had problems with her mucosa, and I don’t have much to offer either.*”

Michael, PwPD (age 82, couple 4)

### 3.4. Theme: Planning an Uncertain Future

The future was uncertain and unknown. Planning was difficult, not knowing how the disease would progress. For others, planning was exactly the right thing to do to feel more at ease. When talking about the future, the home was described as something sensitive.

“*What will we do when we can’t stay here anymore, for example. This house means so much to me, and to him as well. But he’s thinking that we should move now, before I get too unwell. I can’t imagine moving away from here.*”

Christina, PwPD (age 65, couple 3)

“*It’s just that I know how things will turn out for him, and that worries me, it really does. I can see that he… he’s deteriorating more, and he’s a bit more withdrawn. It’s… it’s hard to see.//Yes, we’ve already started thinking that we might have to leave our house and live in an apartment.*”

Marianne, partner (age 78, couple 4)

*The future doesn’t look very cheerful. We haven’t talked much about it but… I don’t have much to say, neither long-term care nor death seems particularly appealing…//There are these hints, about what will happen later, or about the future—it doesn’t look very good…//But I’ve told my children that she has been the one who has gone with me to the doctor and everything, and she has always helped me and stood by me in all of it. She has taken care of everything for me, and to remember that when I’m dead, when she’s still alive, then you… then you must help her.’ And I’ve said that several times.*”

Stephan, PwPD (age 68, couple 5)

## 4. Discussion

The present study aimed to explore how PD affects the marital relationship from the perspectives of both PwPDs and their partners. The findings highlight that living with PD is not only an individual challenge, but also a shared experience shaped by collaboration, emotional adaptation, and relational negotiation. Even though these couples had been married for many years, managing the disease in partnership meant not only nurturing the relationship but also facing serious marital hardship. The couples strived to maintain normalcy and togetherness while confronting the hardships caused by the disease. PD was viewed as a joint responsibility rather than an individual burden. A positive attitude and pragmatic outlook fostered resilience. Spousal support included both being involved with the partner and allowing independence for both. There was a constant requirement for adaptation and navigation of emotional and physical challenges together. For the partners, having to take over chores that they were not used to doing and the constant worry over the partner with PD meant many compromises and consequences, for example, having to arrange for help if they wanted to leave the house for some hours, or having their sleep affected. For the PwPDs, changes in cognitive and communicative functioning contributed to a perceived loss of meaningful and stimulating conversations that had previously characterized the relationship. This shift increased emotional distance within the partnership and contributed to growing social isolation, particularly as time spent at home increased. Striving to preserve the sense of self that existed prior to diagnosis was highlighted by Haar et al. [23], with a strong desire for PwPDs to remain the person they once were. Adaptation required acceptance, self-understanding, and integrating the realities of living with a chronic condition while negotiating the loss of aspects of the pre-diagnosis self.

Our results demonstrate PD is overwhelming for both members of a couple, especially for partners, who reported emotional responses including anger, sadness, and persistent worry, reflecting the psychosocial strain associated with disease progression. Vatter et al. [24] and Dujardin et al. [25] demonstrate a clear interruption due to PD reducing couples’ relationship satisfaction, intimacy, and communication, leading to more emotional distance. Furthermore, cognitive impairment was significantly harder to accept, manage and cope with than the motor symptoms of PD. According to Wu et al. [26], the transition for the partner can lead to frustration, resentment, anger, sadness, and a higher risk for depressive symptoms. Hand et al. [27] described how this strain on the relationship increases even more in the later stages of the disease. Patients’ mental symptoms were the strongest predictors of partners’ distress. The partners in our study expressed a sense of grief, recognizing that things will never be as they once were and understanding the likely progression of the disease. Carter et al. [28] showed that partners living with a PwPD experience a condition called pre-death grief or prolonged grief, knowing that their spouse is disappearing more as time goes by. Similar results for family carers of persons with dementia have been shown [29].

We are interested in how a relationship can survive hardship and if partners feel a tension between love and duty in the relationship when it is threatened by a life-altering disease. In a study by Martin et al. [30], hardships that couples faced included the shifting of relational roles, changes in sexual intimacy, financial stress, and difficulty engaging in leisure and social activities together. Despite this, couples continued to give and receive care from each other. The disease disrupted the balance of caregiving, but couples adjusted by acknowledging this imbalance and striving to maintain a form of mutual care acceptable to both. The partners in our study still managed to express improvements in aspects of their relationship quality with the help of their positive attitudes and pragmatic problem-solving contributing to resilience and a sense of unity. Some couples could continue with normalcy despite facing the threats that PD imposed on the marital relationship. This is supported by Badr & Acitelli [31] and D’Ardenne [32], who found that partners facing chronic illness can adapt to the demands of the condition and grow together through collaboration. Similarly, Conway et al. [33] showed that resilience involved maintaining a sense of normality despite the disease, thereby strengthening togetherness and reciprocity in the relationship. Previous research on couples facing serious illness described their ability to perceive it as a “we disease,” meaning they confronted it together despite hardships [34]. Habermann et al. [35,36] found that couples facing PD engaged in joint decision-making, managing the disease together and making shared decisions about their present and future. They preferred to remain in their own homes, minimizing changes, and receiving increased home-based support.

The term dyadic coping describes the processes through which partners communicate about stress and how they work together to manage it [37]. It has been associated with better physical health, well-being, and relationship satisfaction among couples facing chronic illness [37,38,39]. However, not all couples perceive illness as a “we disease.” Some experience emotional, physical, and financial strain, which may relate to coping styles and relationship quality [40]. Bodenmann’s theory of dyadic coping [41] suggests that it is positively associated with higher relationship quality and a lower risk of divorce. Conversely, misunderstanding each other’s stress, as well as insults, arguments, and lack of respect for each other’s needs, negatively affects dyadic coping. Our results showed that partners expressed a need for time to themselves to regain energy and attend to their interests and hobbies. The PwPDs facilitated the occurrence of this. McRae et al. [42] showed that partners attending support groups and talking to people in the same situation resulted in them feeling less lonely and more supported and improved their well-being. From the patient’s perspective, the isolation reported by PwPDs may be alleviated through support in engagement in meaningful activities, such as physical activity as part of their treatment plan [43,44]. Music-based therapy has also been recommended as an effective intervention to improve motor function, balance, and mental health in PwPDs [45].

Several studies have emphasized nurses’ supportive role in promoting joint decision-making for couples [34,35] and strengthening communication and togetherness between partners [46,47]. We recommend that specialist nurses, with the patient’s consent, invite both partners to the appointments. Even in long-term marriages, unity should not be assumed to imply strength or resilience. We encourage nurses to ask how the couple manages daily life, ensuring that both the PwPD and the partner can express their needs. This includes discussing desired supportive interventions, offering follow-ups, encouraging both shared and individual activities, and acknowledging the partner’s need for time alone. Specialist nurses should serve as core contacts for patients and families, coordinating closely with the care team. Studies by Hellqvist & Berterö [48] and Fujita et al. [49] confirm that PwPDs and their partners highly value specialist nurses for their level of knowledge on the disease and its impact on daily life, as well as their ability to answer questions, stay up to date with research, and provide continuity of care. This continuity fosters trust, reduces the need to repeat one’s story, and creates security for patients and partners.

Modern healthcare emphasizes person-centered care, prioritizing individualized support and compassionate communication [50]. Effective communication strategies are essential for reducing poor outcomes and building trust [51,52]. Trust forms the foundation of the nurse–patient relationship and enhances the quality of care, clinical reasoning, and decision-making. Nurses gain trust by demonstrating a genuine intent to act in the patient’s best interest, which positively influences interactions with both PwPDs and their partners [53,54].

### 4.1. Clinical Implications Grounded in Findings

The findings suggest that couples living with PD benefit from approaches that recognize the illness as a shared experience rather than an individual condition. Nursing interventions that strengthen joint coping strategies and support reciprocal communication can help couples better navigate the disease despite the challenges of disease progression. Nurses should therefore consider involving both partners in education, counseling, and support initiatives. At the same time, nurses should recognize signs of relational distress, such as communication avoidance, loneliness, or increased partner workload, which may suggest that healthcare providers provide couple-based support. Table 4 presents examples of clinical implications related to these themes and subthemes.

### 4.2. Strengths and Limitations

This study makes an important contribution by offering detailed insight into the relational challenges faced by couples living with PD, drawing on data from both PwPDs and their partners. The homogenous background of the participants may be considered a limitation of this study. All participants were elderly (65 years or older), retired, and of Swedish origin without language barriers. Furthermore, they were married long-term (31–60-year relationship) and therefore had experience with coping strategies for marital hardships. These characteristics may limit the transferability of the findings to other PD contexts, such as younger, working, or childbearing couples, whose experiences may differ. However, this concern is partly mitigated by the fact that PD is most often diagnosed in individuals aged 60 years or older.

Three of the couples were recruited via an advertisement in the journal of the Swedish Parkinson’s Disease Association. The purpose of that was to broaden the recruitment to the whole country and not only to the clinic in the metropolitan region of Stockholm. It could be argued that this added a self-selection bias into the sample with persons who are well-functioning and willing to share relational experiences. A strength was the individual interviews, which provided the participants with the opportunity to freely express their feelings and experience of the disease and relational issues without fear of offending their partner. Even if the individual in-depth interviews led to rich data in the present study, some participants might have been uncomfortable expressing relational issues. However, the participants seemed eager to share their experience and coping strategies. During the time interviews were conducted, no major changes occurred in Parkinson’s disease care pathways, specialist nurse roles, or management practices in Sweden. Therefore, the time span of data collection was not impacted by these. During analysis, attention was paid to outlier cases. For example, one couple described detailed and highly specific aspects of their sexual relationship; however, as this was an isolated case and involved sensitive information, it was not included in the results to protect confidentiality and because it did not contribute to the development of shared themes. Finally, we would like to mention that this study was part of a larger project that initially aimed to include individuals with Parkinson’s disease with cognitive impairment, as stated in the information sheet. However, as recruitment progressed, no participants with cognitive impairment were included. Given that sufficient data saturation and depth were achieved with the interviews conducted, and due to limited financial resources, we did not pursue further recruitment targeting this group. We acknowledge the resulting inconsistency between Appendix A and Appendix B and the manuscript; however, Appendix A and Appendix B reflect the initial study design.

### 4.3. Future Research

Although studies have explored how couples manage Parkinson’s disease, there remains a need for longitudinal dyadic studies following couples over time, which would provide further valuable insights into their shared experiences and adaptations. In addition, gaps remain regarding cultural differences in caregiving roles in the context of Parkinson’s disease. Future research could also benefit from action-oriented approaches, such as targeted nursing interventions for couples, including practical communication training.

## 5. Conclusions

This study highlights that Parkinson’s disease is not only a medical condition but also a shared life-altering experience that affects marital relationships. The findings show how couples navigate the emotional, practical, and relational consequences of PD through continuous adaptation, negotiation, and mutual support. Managing the disease together in partnership emerged as a central strategy, with positive attitudes and pragmatic problem-solving contributing to a sense of unity. At the same time, the study reveals significant marital hardships. Shifting roles and responsibilities often placed increased demands on partners, leading to emotional distress and a desire for solitude for the partner. Feelings of grief emerged over the loss of the life they had once envisioned, along with an understanding of the disease’s future progression. Communication played a crucial role: reciprocal and open dialogue brought closeness and understanding, while avoidance and lack of mutual understanding intensified emotional distance and loneliness. The findings have clear implications for healthcare practice. Nurses and other healthcare professionals should include both the PwPD and their partner in designing care and providing support. Specialist nurses can play a crucial role by offering continuity, disease-specific knowledge, emotional support, and guidance tailored to the couple’s needs.

## Figures and Tables

**Table 1:** Demographics of the couples.

Pseudonym PwPD	Stage *	Age	Years with PD	Pseudonym Partner	Age	Years in Relationship
Eric, PwPD (age 69, couple 1)	4	69	15	Cecilia, partner (age 66, couple 1)	66	40
Marie, PwPD (age 69, couple 2)	4	69	15	Thomas, partner (age 70, couple 2)	70	36
Christina, PwPD (age 65, couple 3)	4	65	15	Alfred, partner (age 70, couple 3)	70	47
Michael, PwPD (age 82, couple 4)	4	82	28	Marianne, partner (age 78, couple 4)	78	53
Stephan, PwPD (age 68, couple 5)	4	68	7	Helen, partner (age 75, couple 5)	75	31
Carl, PwPD (age 75, couple 6)	5	75	10	Susanne, partner (age 75, couple 6)	75	60

* According to Hoehn & Yahr, 1967 [19].

**Table 2:** Examples of the coding process for the transcripts.

Quotation	Meaning Unit	Code	Subtheme	Theme
“I’m a positive person and feel like, ‘Yeah yeah, we’ll sort this out. What will be will be. We’ll make the best of it.’ Then it’s much easier to… it sounds strange, but it’s much easier to handle everyday life with that attitude than to wake up in the morning and think everything is awful.” Christina, PwPD	We’ll make the best of it.	Coping strategies	A positive attitude towards life	Managing the disease together in partnership
“I feel stressed all the time, I can’t relax, and I feel a strong need to be alone. But I’m not alone as much as I want to be.” Helen, partner	I’m not alone as much as I want to be.	Own time	Partners’ desire for solitude	Facing marital hardship

**Table 3:** Themes, subthemes and examples of quotation.

Theme	Subtheme	Quotation
Managing the disease together in partnership	A positive attitude towards life	“If you were to get depressed, that wouldn’t help much. It doesn’t solve any problems. It’s better to look on the bright side.” Carl, PwPD
	Having a pragmatic outlook on life	“Yep, we just had to get on with it. And that’s what we’ve done our whole lives with everything. ‘Alright, this is how it is. Then we’ll do it this way.’” Susanne, partner
	Providing support and enabling personal time for their partner	“But it’s very important that she gets to go on trips and experience things that I can’t take part in. Without it being interpreted as some sort of threat to our relationship.” Eric, PwPD
Nurturing the relationship	Doing activities together	“We are reading a book aloud together, for example, taking turns and then talking about it. Yes, so… I feel that we talk much more with each other now than we used to.” Thomas, partner
	Reciprocal communication brings closeness in the relationship	“We can be sad together and we can laugh a lot together, and we have the same kind of humor, so yes, it works, it works well… you could say that it has become a bit deeper.” Alfred, partner
Facing marital hardship	Shifting relational roles can lead to emotional distress	“I had to keep an eye on him to make sure he took his medication. He started forgetting that too. Suddenly I had to shoulder much more. And it became stressful for me to keep track of him all the time.” Cecilia, partner
	Partners’ desire for solitude	“I really feel a strong urge to be by myself a lot.” Cecilia, partner
	Lack of reciprocal communication and mutual understanding	“When you’re struggling a bit and are lying awake at night unable to sleep, it’s not something you can just shake off the way you once could. It’s hard, but I can’t go and wake her up to start talking about it. No, no—then you’re alone with your thoughts.” Stephan, PwPD
	Loss of what once was	“We have our age against us, both of us. I talk to the nurse about it, that sex has faded away. My partner had problems with her mucosa, and I don’t have much to offer either.” Michael, PwPD
Planning an unknown future		“What will we do when we can’t stay here anymore, for example. This house means so much to me, and to him as well. But he’s thinking that we should move now, before I get too unwell. I can’t imagine moving away from here.” Christina, PwPD

**Table 4:** Clinical implications grounded in findings.

Theme/Subtheme	Key Findings	Clinical Implications
Managing the disease together in partnership—A positive attitude towards life and having a pragmatic outlook.	A shared positive outlook helped couples cope with daily challenges and maintain emotional resilience. Couples often used practical problem-solving strategies to adapt to disease-related changes.	Healthcare professionals can provide couple-focused support, encouraging the partner to come to the visits. Attending together may strengthen their practical coping strategies and collaborative problem-solving skills.
Managing the disease together in partnership—Providing support and enabling personal time for their partner	Some partners experienced stress and a need for solitude, indicating emotional strain. Partners needed opportunities for time alone to sustain caregiving and relationship balance.	Health care services should facilitate opportunities and encourage partners to maintain personal activities and social support. Identifying caregiver stress and offering psychosocial support may help prevent relational burnout.
Nurturing the relationship—Doing activities together	Shared activities strengthened couples’ connection and helped couples maintain their togetherness.	Couples may benefit from guidance on adapting meaningful shared activities as functional abilities change over time.
Nurturing the relationship—Reciprocal communication brings closeness in the relationship	Open communication fostered closeness and helped couples navigate emotional challenges.	Couple-based communication support or counseling may help partners express needs and maintain emotional connection.
Facing marital hardship—Shifting relational roles	Changes in responsibilities increased partner workload and sometimes led to emotional strain.	Nurses should assess caregiver burden and provide support or resources to partners taking on increasing responsibilities.
Planning an uncertain future	Couples differed in their willingness to discuss future care and living arrangements.	Nurses can facilitate sensitive conversations about future planning and long-term care preferences.

## Data Availability

Data (tape-recorded interviews and transcripts) is unavailable due to privacy and ethical restrictions to maintain confidentiality and protect the participants’ identities.

## Public Involvement Statement

No public involvement in any aspect of this research.

## Guidelines and Standards Statement

This manuscript was drafted with reference to The Standards for Reporting Qualitative Research (SRQR) for qualitative research [55].

## Abbreviations

The following abbreviations are used in this manuscript:

PD	Parkinson’s disease
PwPD	Person with Parkinson’s disease

## Appendix A

### Interview Guide

- Can you tell me what it was like when you first began to notice symptoms?
- How did you experience that period of time?
- How did you react when you were diagnosed?
- Were you able to share this with anyone? With whom?
- How much were you able to talk about it?
- In what ways do you feel your life has changed since your diagnosis?
- How do you feel your partner’s life has changed?
- Is there anything you find difficult or challenging to share with others?
- How has your relationship changed?
- Have your daily activities changed in any way?
- How are household tasks managed?
- How do you communicate with each other?
- How do you handle challenges that you face because of Parkinson’s disease?
- How do you view the future?
- What hopes do you have?
- What concerns or fears do you have?
- Do you receive any support or help from others?
- What kind of support do you currently receive?
- What support would you like to have?

## Appendix B

### Informed Consent

**Impact on the Couple Relationship in Parkinson’s Disease and Cognitive Impairment**

You are hereby invited to participate in a research project with the aim of investigating how Parkinson’s disease and mild cognitive impairment affect the couple relationship. We invite participants to the research project via the Neurology and Memory Clinic at Karolinska University Hospital, Huddinge. Read the information below about the project and feel free to ask questions if you have any questions.

**What is the background and purpose of the study?**

In Parkinson’s disease, motor symptoms, so-called movement-related symptoms such as tremors, stiffness and slow movements, are typical symptoms, but non-motor symptoms such as depression, anxiety and sleep disorders, loss of smell and taste and constipation are also common. Some of those who have Parkinson’s disease but also people without Parkinson’s disease may experience that their thinking and memory skills are affected, so-called cognitive impairment, which means that they may experience difficulties learning new things and understanding instructions and this in turn can affect how well they cope with different situations in daily life. International studies have shown that these symptoms can to varying degrees negatively affect the couple’s relationship and the health-related quality of life, and that relatives who are involved in the care of those affected experience increased stress and strain. However, knowledge is still lacking among patients, relatives and healthcare professionals about how these symptoms affect the couple’s relationship, the experience of strain and quality of life. With this study, we want to gain a deeper understanding and shed light on how the couple relationship is affected when someone has Parkinson’s disease and, if so, how the couple relationship changes over time, to be able to identify the support you need. We hope that the results of this study will form the basis for development and improvement work in the care and welfare of families affected by Parkinson’s disease and cognitive impairment.

**How is the study conducted?**

The present study is a longitudinal interview study that will include 20 couples, one of whom has Parkinson’s disease. Those who participate will be interviewed on two occasions over a two-year period. The interviews will strive to take the form of an open dialogue with open-ended questions with the aim of exploring different aspects of your experiences and perceptions and will take place individually with you and your life partner in a separate room without disturbances. The interviews are expected to take approximately 1 h and will be recorded on a tape recorder so that they can then be transcribed verbatim and analyzed. The study is planned by doctors and researchers at Karolinska Institutet in collaboration with Karolinska University Hospital, Huddinge. The Ethics Review Board in Stockholm has reviewed and approved the ethical issues of the study.

**What does participation in the study entail?**

Your printed story will be treated confidentially so that no unauthorized persons can access it and will only be used in the research project. The printed story will also be coded so that neither your name nor your social security number will be used when the story is analyzed. The code key, recorded material and printed stories will be stored in a locked space that meets Karolinska Institute’s criteria for storing research material. The Privacy Act, the Health Care Register Act and the Personal Data Act protect your personal data. Analysis and reporting of the study results will take place at the group level, which means that no information that can identify you as an individual or business will be published.

**Will my participation pose any risks to me?**

Participating in interviews can raise emotions and concerns. If you as a study participant have concerns/questions or experience any form of concern during the study period, you are welcome to contact the doctors and researchers listed below.

Karolinska University Hospital, Huddinge

Johan Lökk (Principal investigator)

Information: telephone +46 (0)8 58580000

I Principal investigator, texted have explained the study design and purpose to (patient and partner’s name, texted)

.........................................................................................................................................................

………………………………………………………………………………………………………
Signature of principal investigator Date

Karolinska Institutet

**Informed consent**

I confirm that I have received this written and other oral information about the research study and have had the opportunity to discuss its content with the principal investigator.

I give my consent to participate in the study and know that my participation is completely voluntary.

I am aware that I can end my participation at any time and without explanation, and this will not affect any future care and treatment.

As a patient, I have been informed and I allow that my personal data is noted in a medical record and that collected data and results about me will be stored and administrated electronically by the responsible persons of the study.

This may be done with the proviso that the information that becomes available there is not passed on.

As a spouse/partner, I have been informed and I allow that my personal data, collected data and results about me will be stored and administrated electronically by the responsible persons of the study.

This may be done with the proviso that the information that becomes available there is not passed on.

..............................................................................................................................
Patient’s signature Date

..............................................................................................................................
Spouse’s/Partner’s signature Date

## References

[1] GBD 2016 Parkinson’s Disease Collaborators (2018). Global, regional, and national burden of Parkinson’s disease, 1990–2016: A systematic analysis for the Global Burden of Disease Study 2016. Lancet Neurol..

[2] Ascherio A., Schwarzschild M.A. (2016). The epidemiology of Parkinson’s disease: Risk factors and prevention. Lancet Neurol..

[3] Armstrong M.J., Okun M.S. (2020). Diagnosis and Treatment of Parkinson Disease: A Review. JAMA.

[4] Jankovic J. (2008). Parkinson’s disease: Clinical features and diagnosis. J. Neurol. Neurosurg. Psychiatry.

[5] DeMaagd G., Philip A. (2015). Parkinson’s Disease and Its Management: Part 1: Disease Entity, Risk Factors, Pathophysiology, Clinical Presentation, and Diagnosis. Pharm. Ther..

[6] Chaudhuri K.R., Odin P., Antonini A., Martinez-Martin P. (2011). Parkinson’s disease: The non-motor issues. Park. Relat. Disord..

[7] Caillava-Santos F., Margis R., de Mello Rieder C.R. (2015). Wearing-off in Parkinson’s disease: Neuropsychological differences between on and off periods. Neuropsychiatr. Dis. Treat..

[8] Aderinola O.M., Ozojide K.O., Nwachukwu E.M., Okobi O.E., Eneh V., Mbonu J.C., Abah O.C., Bakare T., Aniagbaoso E.A., Singh G. (2025). Assessment of Health-Related Quality of Life in Parkinson’s Disease: A Systematic Review. Cureus.

[9] Bhimani R. (2014). Understanding the Burden on Caregivers of People with Parkinson’s: A Scoping Review of the Literature. Rehabil. Res. Pract..

[10] Aamodt W.W., Kluger B.M., Mirham M., Job A., Lettenberger S.E., Mosley P.E., Seshadri S. (2024). Caregiver Burden in Parkinson Disease: A Scoping Review of the Literature from 2017–2022. J. Geriatr. Psychiatry Neurol..

[11] Lökk J. (2009). Reduced life-space of non-professional caregivers to Parkinson’s disease patients with increased disease duration. Clin. Neurol. Neurosurg..

[12] Longacre M.L., Roche L., Kueppers G.C., Buurman B. (2025). Parkinson’s Disease and Caregiving Roles, Demands, and Support Needs and Experiences: A Scoping Review. Healthcare.

[13] Goldsworthy B., Knowles S. (2008). Caregiving for Parkinson’s disease patients: An exploration of a stress-appraisal model for quality of life and burden. J. Gerontol. Ser. B Psychol. Sci. Soc. Sci..

[14] Archbold P.G., Stewart B.J., Greenlick M.R., Harvath T. (1990). Mutuality and preparedness as predictors of caregiver role strain. Res. Nurs. Health.

[15] Karlstedt M., Fereshtehnejad S.M., Aarsland D., Lökk J. (2017). Determinants of Dyadic Relationship and Its Psychosocial Impact in Patients with Parkinson’s Disease and Their Spouses. Park. Dis..

[16] Tanji H., Anderson K.E., Gruber-Baldini A.L., Fishman P.S., Reich S.G., Weiner W.J., Shulman L.M. (2008). Mutuality of the marital relationship in Parkinson’s disease. Mov. Disord..

[17] Karlstedt M., Fereshtehnejad S.-M., Dag Aarsland D., Lökk J. (2018). Mediating Effect of Mutuality on Health-Related Quality of Life in Patients with Parkinson’s Disease. Park. Dis..

[18] Glover L., Dixon C., Kobylecki C., Eccles J.R. (2023). Parkinson’s and the couple relationship: A qualitative meta-synthesis. Aging Ment. Health.

[19] Hoehn M.M., Yahr M.D. (1967). Parkinsonism: Onset, progression and mortality. Neurology.

[20] Brinkmann S. (2013). Qualitative Interviewing. Understanding Qualitative Research.

[21] Hsieh H.-F., Shannon S.E. (2005). Three approaches to qualitative content analysis. Qual. Health Res..

[22] Ahmed S.K. (2024). The pillars of trustworthiness in qualitative research. J. Med. Surg. Public Health.

[23] Haahr A., Groos H., Sørensen D. (2021). ‘Striving for normality’ when coping with Parkinson’s disease in everyday life: A metasynthesis. Int. J. Nurs. Stud..

[24] Vatter S., McDonald K.R., Stanmore E., Clare L., McCormick S.A., Leroi I. (2018). A qualitative study of female caregiving spouses’ experiences of intimate relationships as cognition declines in Parkinson’s disease. Age Ageing.

[25] Dujardin K., Manceau C., Wawrziczny E., Flinois B., Baille G., Defebvre L., Antoine P. (2025). When Parkinson’s disease interferes in a couple’s life: A qualitative study. J. Park. Dis..

[26] Wu J., Liu M., Chen M., Xue J., Zou Y., Deng Z., Zhao S., Yang X. (2025). Factors associated with higher caregiver burden among informal caregivers of Parkinson’s disease: A systematic review. Medicine.

[27] Hand A., Oates L.L., Gray W.K., Walker R.W. (2019). The role and profile of the informal carer in meeting the needs of people with advancing Parkinson’s disease. Aging Ment. Health.

[28] Carter J.H., Lyons K.S., Lindauer A., Malcom J. (2012). Pre-death grief in Parkinson’s caregivers: A pilot survey-based study. Park. Relat. Disord.

[29] Moore K.J., Crawley S., Fisher E., Cooper C., Vickerstaff V., Sampson E.L. (2023). Exploring how family carers of a person with dementia manage pre-death grief: A mixed methods study. Int. J. Geriatr. Psychiatry.

[30] Martin S.C. (2016). Relational Issues Within Couples Coping with Parkinson’s Disease: Implications and Ideas for Family-Focused Care: Implications and Ideas for Family-Focused Care. J. Fam. Nurs..

[31] Badr H., Acitelli L.K. (2005). Dyadic Adjustment in Chronic Illness: Does Relationship Talk Matter?. J. Fam. Psychol..

[32] D’Ardenne P. (2004). The couple sharing long-term illness. Sex Relatsh. Ther..

[33] Conway L., Wolverson E., Clarke C. (2020). Shared Experiences of Resilience Amongst Couples Where One Partner Is Living with Dementia-A Grounded Theory Study. Front. Med..

[34] Kayser K., Watson L.E., Andrade J.T. (2007). Cancer as a “we-disease”: Examining the process of coping from a relational perspective. Fam. Syst. Health.

[35] Habermann B., Shin J.Y. (2017). Preferences and concerns for care needs in advanced Parkinson’s disease: A qualitative study of couples. J. Clin. Nurs..

[36] Habermann B., Shin J.Y., Shearer G. (2020). Dyadic Decision-Making in Advanced Parkinson’s Disease: A Mixed Methods Study. West J. Nurs. Res..

[37] Bertschi I.C., Meier F., Bodenmann G. (2021). Disability as an Interpersonal Experience: A Systematic Review on Dyadic Challenges and Dyadic Coping When One Partner Has a Chronic Physical or Sensory Impairment. Front. Psychol..

[38] Weitkamp K., Feger F., Landolt S.A., Roth M., Bodenmann G. (2021). Dyadic Coping in Couples Facing Chronic Physical Illness: A Systematic Review. Front. Psychol..

[39] Muijres P., Bodenmann G., Nussbeck F.W., Jenewein J. (2025). Dyadic coping and well-being in early-stage dementia couples. Front. Psychiatry.

[40] Zhang A., Mitchell L.K., Pachana N.A., Yang J.H.J., Au T.R., Byrne G.J., O’Sullivan J.D., Dissanayaka N.N. (2022). Caring for a loved one with Parkinson’s disease: The role of coping styles and relationship quality. Int. Psychogeriatr..

[41] Bodenmann G., Revenson T.A., Kayser K., Bodenmann G. (2005). Dyadic Coping and Its Significance for Marital Functioning. Couples Coping with Stress: Emerging Perspectives on Dyadic Coping.

[42] McRae C., Fazio E., Hartsock G., Kelley L., Urbanski S., Russell D. (2009). Predictors of loneliness in caregivers of persons with Parkinson’s disease. Park. Relat. Disord..

[43] Van Nimwegen M., Speelman A.D., Hofman-van Rossum E.J., Overeem S., Deeg D.J., Borm G.F., van der Horst M.H., Bloem B.R., Munneke M. (2011). Physical inactivity in Parkinson’s disease. J. Neurol..

[44] Langeskov-Christensen M., Franzén E., Grøndahl Hvid L., Dalgas U. (2024). Exercise as medicine in Parkinson’s disease. J. Neurol. Neurosurg. Psychiatry.

[45] Zhou Z., Zhou R., Wei W., Luan R., Li K. (2021). Effects of music-based movement therapy on motor function, balance, gait, mental health, and quality of life for patients with Parkinson’s disease: A systematic review and meta-analysis. Clin. Rehabil..

[46] Manceau C., Constant E., Brugallé E., Wawrziczny E., Sokolowski C., Flinois B., Baille G., Defebvre L., Dujardin K., Antoine P. (2023). Couples facing the “honeymoon period” of Parkinson’s disease: A qualitative study of dyadic functioning. Br. J. Health Psychol..

[47] Martire L.M., Helgeson V.S. (2017). Close relationships and the management of chronic illness: Associations and interventions. Am. Psychol..

[48] Hellqvist C., Berterö C. (2015). Support supplied by Parkinson’s disease specialist nurses to Parkinson’s disease patients and their spouses. Appl. Nurs. Res..

[49] Fujita T., Iwaki M., Hatono Y. (2024). The role of nurses for patients with Parkinson’s disease at home: A scoping review. BMC Nurs..

[50] Phelan A., McCormack B., Dewing J., Brown D., Cardiff S., Cook N., Dickson C., Kmetec S., Lorber M., Magowan R. (2020). Review of developments in person-centred healthcare. Int. Pract. Dev. J..

[51] Afriyie D. (2020). Effective communication between nurses and patients: An evolutionary concept analysis. Br. J. Community Nurs..

[52] Pitts E., Wylie K., Loftus A.M., Cocks N. (2022). Communication strategies used by Parkinson’s nurse specialists during healthcare interactions: A qualitative descriptive study. J. Adv. Nurs..

[53] Bell L., Duffy A. (2009). A concept analysis of nurse-patient trust. Br. J. Nurs..

[54] Carter M.A. (2009). Trust, power, and vulnerability: A discourse on helping in nursing. Nurs. Clin. N. Am..

[55] O’Brien B.C., Harris I.B., Beckman T.J., Reed D.A., Cook D.A. (2014). Standards for reporting qualitative research: A synthesis of recommendations. Acad Med..

